# Supplementary material for: Older Adults Who Maintained a Regular Physical Exercise Routine before the Pandemic Show Better Immune Response to Vaccination for COVID-19
Source: Int J Environ Res Public Health. 2023 Jan 20;20(3):1939. doi: 10.3390/ijerph20031939 (PMC9915291; doi:10.3390/ijerph20031939)
Supplement: Supplementary file 1 [file ijerph-20-01939-s001.zip › ijerph-2076853-supplementary.pdf]

Supplementary material

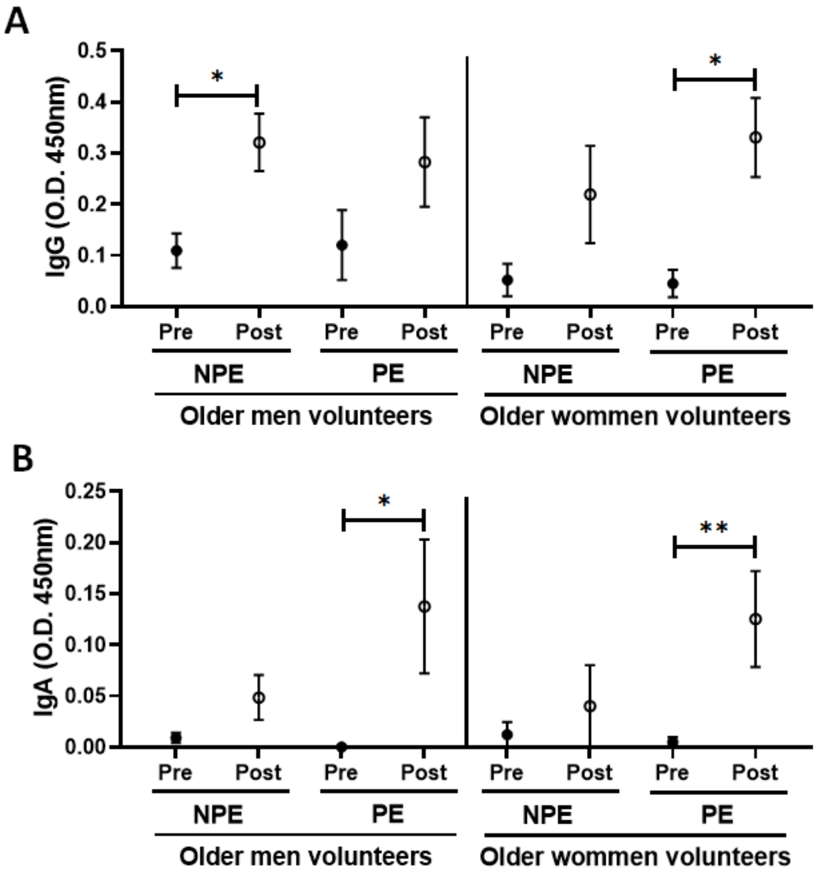

Supplementary Figure S1. Total serum concentration (O.D. 450nm) of specific IgG (A) and IgA (B) for SARS-CoV-2 antigens before (pre) and after 30 days (post) of administration of the second dose of ChadOx-1 vaccine in the subgroups of older men and older women who regularly practiced (PE) or not (NPE) a physical exercise program before the pandemic period. Data are presented as median and interquartile range. *p<0.05; **p<0.01.
